# Supplementary material for: High-Temperature Polylactic Acid Proves Reliable and Safe for Manufacturing 3D-Printed Patient-Specific Instruments in Pediatric Orthopedics—Results from over 80 Personalized Devices Employed in 47 Surgeries
Source: Polymers (Basel). 2024 Apr 26;16(9):1216. doi: 10.3390/polym16091216 (PMC11085401; doi:10.3390/polym16091216)
Supplement: Supplementary file 1 [file polymers-16-01216-s001.zip › Document S1.pdf]

Checklist for PSI control

Virtual Surgical Planning environment (check with surgeon)

1. Verification of the surface area for placing the PSI
2. Verification of positioning and directions of the planned K-wires
3. Verification of positioning and directions of the planned cutting planes

CAD environment

1. Verification of the creation of all necessary holes for the planned K-wires
2. Verification of the creation of all necessary saw blade slots corresponding to the planned cutting planes
3. Verification of the absence of sharp edges, the entire model must be smoothed

Slicer environment – 3D-pinting preparation

1. Verification of placing the cutting guide on the printing bed with the surface intended for bone contact facing upward to prevent damage from supports
2. Check for proper placement on the printing bed to ensure no supports are within the holes and saw blade slots
3. Monitoring the printing layer progression to ensure no areas with model reading errors
4. Monitoring the printing layer progression to ensure all protruding areas are supported

Real environment – 3D-printed performed

1. Verification that the 3D-printed part has completed the 3D-printing process entirely
2. Removal of the part from the printing bed and visual inspection of the part compared to the CAD model
3. Delicate removal of supports, taking care not to damage the part
4. Checking the passage of K-wires and saw blades
5. Checking the fit of the cutting guide, holes, and saw blade slots on a previously 3D-printed patient-specific bone with reference to K-wire and cutting planes positioning

Real environment – Heat treatment performed

1. Visual inspection for analysis of any significant thermal deformations
2. Checking the passage of K-wires and saw blades
3. Post-treatment cutting guide fit check for deformation analysis. Re-checking the fit of the cutting guide, holes, and saw blade slots holes on the 3D-printed patient-specific bone used in point 5 of the previous section
4. Documentation check of the material technical data sheet for delivering the PSIs to the sterilization centre
