# Supplementary material for: High-Temperature Polylactic Acid Proves Reliable and Safe for Manufacturing 3D-Printed Patient-Specific Instruments in Pediatric Orthopedics—Results from over 80 Personalized Devices Employed in 47 Surgeries
Source: Polymers (Basel). 2024 Apr 26;16(9):1216. doi: 10.3390/polym16091216 (PMC11085401; doi:10.3390/polym16091216)
Supplement: Supplementary file 1 [file polymers-16-01216-s001.zip › Document S2.pdf]

Management of 3D MALF II Medical Devices at the sterilization facility.

The Central Office operator, by means of management software equipped with an optical reader, handles the collection of medical devices that are delivered directly from the pharmacy, recording their entry, characteristics, treatment methods as well as any notes pertaining to the device itself and its use. After registration and collection, the medical device is brought into the operational areas of the facility to undergo the sterilization process and the preliminary steps related to it.

Generalities

At the Sterilization Facility, there is specific equipment, such as washer-disinfectors, thermosealers and autoclaves, which are necessary for the implementation of the sterilization process and which are validated annually as indicated in UNI EN ISO 17665-1 and ISO/TS 17665-2.
To perform monitoring of the sterilization process, three different controls are available: physical controls, performed daily and before the sterilization activity to verify the operation of autoclaves, chemical controls as process and outcome indicators, and biological controls.

The following table shows the type and frequency of monitoring, the indicators used, and the filing mode

CONTROLS	INDICATORS	FREQUENCY	RECORDING
**Physical**	✓ Thermometer<br>✓ Manometer<br>✓ Acoustic alarm<br>✓ Print recording	At each sterilization cycle	Graphs and parametric releases to be attached to the daily autoclave management sheet
**Chemicals**	Process indicators	<u>External</u> to each package	
**Biological**	Geobacillus Stearothermophilus (ATCC 7953)	- Daily (134°C)<br>- Weekly (121°C)<br>- After routine or extraordinary maintenance work on the indication of the technician performing the maintenance work	Receipt issued directly from the biological evidence reader/incubator attached to the registration document
**Preheating ***<br>(preliminary cycle of vacuum sterilization)		Daily (before autoclave use in case the autoclave was turned off overnight or weekend)	Daily autoclave management sheet
**Vacuum Test (VT)***<br>(Tightness test of the sterilization chamber)	Allowable leakage is 1.3 mBar/minute	Daily (before autoclave use)	Daily autoclave management sheet
**Bowie-Dick (BD)***<br>(Steam penetration test)	Chemical in ready package	Daily (before autoclave use)	Daily autoclave management sheet
**Elix Test**<br>(Steam penetration test inside hollow bodies)	Chemical with appropriate aid	Daily (before use of autoclave)	Daily autoclave management sheet
**Validation**	- Thermometric Control<br>- Residual Humidity (RH) test ≤ 1.2 % Initial Weight	-Annual<br>-For each individual medical device	-Validation documents<br>-Check list

	(10 kg)		

The first operator on duty in the morning performs autoclave warm-up (if the machine had been turned off overnight), leakage (or leakage) test, Bowie & Dick test, Helix Test, and Biological test.

The Bowie & Dick test is a disposable ready pack, has easy and quick use, and is used daily to check the proper functioning of the air removal system in the sterilizer.
It consists of two blocks of porous paper material and an indicator sheet inserted in the center of the two blocks of porous material.
Since the chemical indicator foil spoil ink is easily deteriorated, the product should be stored at room temperature (between 0 and 30 degrees Celsius), away from light and at relative humidity conditions between 20 and 60 percent.
Batch and expiration data are recorded on software and stock checked by RC or his designated operator upon arrival at the plant.
Proper operation of the sterilizer is evidenced by the uniform coloring of the sheet from light blue to pink. Instructions for the proper use of this equipment are available in Instruction No. 4 (Bowie & Dick Test Instructions).
The possible presence of yellow/brown or gray/silver stains indicates a failed test; to identify the extent and severity of the problem, consult the information signs provided by the same company.

The Operator records the result of the Bowie & Dick test and files the tacked sheet in the appropriate binder which will then be archived.
Disposal of the material used does not present particular difficulties as it does not give rise to toxic or harmful waste

The Helix Test consists of a cannula and a blue indicator vizing strip and is used daily to check steam penetration inside all hollow bodies.
The correct operation of the autoclave is indicated by the uniform vizing of the strip from light blue to black.
Instructions for the correct use of this presidium are available in the appropriate Instruction No. 5.
For full traceability of batches and deadlines for both tests, our management software provides for recording and archiving of test results in the special "start up" mask filled in by the operator in charge.
It is OC's duty to record in the same "start up" mask daily the batches and expiration dates of the detergents in use, if this mask is not filled in the same management software does not allow the start of the activity.
Again, the disposal of the material used does not present particular difficulties since it does not give rise to toxic or harmful waste.

Regarding the **Leakage Test** please refer to Instruction No. 6 (Leakage test).
Biological Test consists of a vial containing heat-resistant spores that, after undergoing a steam sterilization cycle, are placed in contact with the culture liquid and incubated.
The Simulator is a small cylindrical metal container inside which the indicator for biological control of steam sterilization cycles is placed. The indicator is to be placed inside a silicone tube that protects its integrity from possible shocks and direct contact with metal.
On the bottom of the simulator there is a 0.025 mm diameter hole that makes steam penetration particularly difficult, thus recreating the extreme conditions that can occur inside containers.
The Simulator allows the biological test to be performed in the autoclave providing additional assurance, given the extreme conditions, that sterilization has taken place.

In the morning, after the tests done by OC, at the first autoclave cycle, proceed to insert the biological indicator inside the silicone tube and enter the data provided by the dedicated Steritrack mask.
Place the Simulator in the cart along with the containers and insert the load into the autoclave.

At the end of the sterilization cycle, the operator removes the biological indicator from the container and incubates it and reads the results as indicated in the dedicated instruction.

Once a week, on Mondays, the operator in charge of the first shift of the washing area performs the test to check the effectiveness of the ultrasonic machine.
Sonocheck is the dosimeter that is used for the routine control of ultrasonic cleaners. The product can indicate whether the machine, during the cleaning cycle, has achieved the correct parameters of delivered energy and cavitation. The test consists of sealed disposable vials containing a green-colored indicator solution

and some cores that are activated in the presence of cavitation. The positioning and reading of the vials are given in Instruction 8.

The operator turns on the washer-disinfectors, if turned off, checking detergent levels, adding them if necessary, and recording on the software's start-up mask batches and expiration dates, as directed by the manufacturer.

The First Shift Worker will also check that the PPE required to perform, with correct clothing, the work activity throughout the day is available in each area.
A person in charge of each area will check that it is properly stocked.
The Steritrack management software in use at the plant provides an initial work opening mask (Start Up) that summarizes the salient functions to be recorded and/or verified and harmonizes them throughout the day to all production batches.

3D MALF II DEVICE PROCESSING

⇒ WASHING

After being registered in the management software, the medical devices in question enter the washing area where they undergo an initial manual washing process aimed at reducing impurities as well as lowering the microbial load carried out through the use of:
1) Ultrasonic Tank
2) Enzymatic cleaner
3) Appropriate brushes
4) High-pressure water guns
5) Medical air guns for drying.

⇒ QUALITY CONTROL AND PACKAGING

Medical devices are then transferred to the packaging area (contamination-controlled environment) through the appropriate pass-through window. Here, the quality checks required by company protocols are carried out by the operators, which include device cleanliness and integrity, and the appropriate BSS (Sterile Barrier Systems) are affixed (DOUBLE BUNDLE).

⇒ STERILIZATION

The packaging stage is followed by the sterilization stage. The process is carried out by means of the autoclave, a machine that uses saturated steam under pressure to perform its microbicidal function.
The parameters chosen are:
Temperature: 134°C
Pressure: 3 Bar
Exposure Time: 7 Minutes

⇒ COOLING, CONTROLS AND TRACKING

Once the sterilization cycle is completed, the medical devices are left to cool in the packaging area, a contamination-controlled environment. Before the sterilization batch is released, all checks are made regarding not only the achievement of the physical parameters of the cycle but also the integrity of the BSS and the possible presence of condensation. If the checks are successful, the accompanying documentation, i.e., all the documentation necessary for traceability of the medical device, is printed. Medical device traceability is managed using Steritrack®, the software developed by Steritalia in collaboration with a software house.
The software, among other things:
→ guides and supports the activities of creating and populating the medical device registry;
→ ensures full traceability of processes by assigning a unique production batch to each DM each time it undergoes reproccessing;
→ supports the activities of operators at every stage of the process by guiding them in the application of the correct treatment protocols.
